# ‘Pressure creates diamonds’/‘fire refines gold’: Conceptualizing coping capital

**DOI:** 10.1007/s13162-022-00236-0

**Published:** 2022-09-01

**Authors:** Vikram Kapoor, Russell Belk

**Affiliations:** 1 Institute of Management Studies, Goldsmiths, University of London, New Cross, SE14 6NW London, UK; 2 Schulich School of Business, York University, 4700 Keele St, Toronto, M3J1P3 Canada

**Keywords:** Consumer coping, Cultural capital, Bourdieu, Resource reservoir

## Abstract

While many consumer behavior studies have investigated consumer coping, few have considered it as a source of positive benefits in addition to being a matter of necessity. In this paper, we draw on Bourdieu’s notion of capital to introduce the concept of coping capital—the intentional or unintentional accumulation of resources, such as emotional and epistemic-competencies and skills resulting from coping with adversity, that *may* thereafter exist in an embodied state in the form of mental and physical dispositions—dispositions that later provide benefits in life. We suggest that the benefits of coping capital may be determined using either a prospective or a retrospective approach. These benefits may be anticipated or unanticipated when intentionally coping with adversity, while the benefits are predominantly unanticipated when unintentionally coping. By conceptualizing coping capital, our study makes a domain-level conceptual contribution to research on consumer coping. In addition the concept of coping capital may have broader implications outside of the domain of consumption.

## Introduction

When faced with adversity, many of us would have heard wise words from our elders, such as “pressure creates diamonds” and “fire refines gold.” Another well-known affirmation of the benefits of challenges is the German philosopher Nietzsche’s aphorism: “What doesn’t kill you, makes you stronger.” The idea of growth from adversities is also present in different world religions. The Christian Bible, for example, reminds us of that, “…we glory in tribulations also: knowing that tribulation worketh patience; and patience, experience; and experience, hope” (Romans 5: 3–4, KJV).

We all need to cope with various stressful situations and predicaments in our lives, such as bereavement, homelessness, old age, addiction, challenges posed by physical and mental illnesses, epidemics and natural catastrophes, unfavorable social environments, domestic violence and abuse, financial hardship, and a variety of other adversities. Considering the pervasiveness of this phenomenon, the topic of coping with stress and adversity has been widely investigated across several disciplines such as psychology (Nelson et al., 2001), sociology (Gutiérrez, 1994), anthropology (Hadley & Crooks, 2012), and marketing and consumer research (Luce, 1998). The phenomenon of consumer coping has become even more consequential in recent times of the COVID-19 pandemic. Consumers’ use of different mediating technologies to maintain a sense of community and social connectedness during the crisis (Kirk & Rifkin, 2020); their use of reappraisal as a coping mechanism, in which they alter their dispositions on the nature of the danger and de-emphasize its intensity (Smith et al., 2021); their meaning-making coping strategy involving people’s selfless acts (August & Dapkewicz, 2020); and their turning to religion and putting faith in God to alleviate their psychological suffering and improve overall well-being (Pirutinsky et al., 2020; Bentzen, 2020) are only a few examples of coping with the ongoing COVID-19 crisis.

Even before this dangerous outbreak, the “highly complex and nuanced” topic of consumer coping (Duhachek 2005, p. 52) had received sustained attention in marketing and consumer research (e.g., Yi & Baumgartner 2004; Saintives & Lunardo, 2016; Jeong & Drolet, 2014; Duhachek & Kelting, 2009; Duhachek & Iacobucci, 2005; Chang & Arkin, 2002). Several studies have examined consumer’s coping strategies (e.g., Viswanathan et al., 2005; Gelbrich, 2010) and resources (e.g., Machin et al., 2019; Yurdakul & Atik, 2015). Nonetheless, a careful reflection on the existing coping scholarship reveals that few have considered coping as a source of positive benefits rather than a matter of necessity. Put differently, the aspect of coping-related growth has yet to be adequately appreciated and given sufficient attention. This, in part, motivated us to conceptualize coping capital. We paid close attention to MacInnis’ (2011) caution that “without conceptualizing new constructs, we would study the same constructs over and over again, limiting our perspectives on the world” (p. 141). Similarly, an excessive focus on consumers’ coping strategies and responses can blind us to the potential long-term benefits of coping with stress and adversity in future circumstances and contexts.

Noting the complicated linkages that comprise society, Merton (1936) contends that the consequences of our actions are not limited to any particular area of concentration; they occur in several associated domains that we entirely ignored while making the action (p. 903). While we do not rule out the possibility that some things may have exactly the opposite effects of the intent with which they were designed, which Tenner (1997) referred to as “revenge effects” in the case of technology, we propose that similar reasoning to Merton applies to our coping with stresses, which may result in unforeseen and beneficial outcomes in a variety of related but previously unconsidered spheres of our lives. Bearing that in mind, our paper tries to answer the research question: How can coping with adversity generate resources that provide benefits to the individual—benefits that are transferable to various contexts of life? In other words, how can coping with difficulties be viewed as capital?

In order to answer the above questions, we conceptualize the notion of coping capital. In our conceptualization, we are less concerned with coping strategies per se, which constitute the majority of the existing consumer coping research. Instead, we focus on how coping with difficulties can generate a reservoir of resources. Hutton’s (2016) article nicely highlights the distinction between coping resources and coping strategies. While coping resources involve factors such as income and education that we draw on in the face of adversity, coping strategies are problem- and emotion-based “recognizable patterns of behavior used to combat stressors” (Hutton, 2016, pp. 253–254). More formally, resources refer to “those objects (e.g., clothing, crystal goblets), conditions (e.g., employment, quality marriage), personal characteristics (carpentry skills, hardiness), and energies (e.g., stamina, knowledge, money) that are valued or that serve as a means of obtaining resources that are valued” (Hobfoll & Freedy, 1993, p. 117). The distinction between coping resources and coping strategies is significant in our conceptualization of coping capital, which we shall see in subsequent sections.

We suggest that coping with adversities can not only help in learning compensatory strategies to employ in the face of specific hardships, but also becomes a source of strength and confidence for facing subsequent challenges in life. That is, we potentially learn strategies and skills to survive tragedies, we also learn that we can come through such trials and tribulations successfully. Therefore, we are concerned less about existing resources that we draw upon, as Hutton suggests, but the new resources generated and the benefits that arise from coping with crisis. The idea that facing adversities generates benefits, e.g., improving self-efficacy and stress resistance, may contribute to health-enhancing lifestyle modifications, engender empathy for others, and lead to finding meaning in life is not new (McMillen, 1999). Rather, the novelty of our conceptualization lies in drawing on Bourdieu to introduce the new concept of coping capital and offering an approach to understanding types of capital in a contemporary consumer culture. Indeed, Bourdieu’s sociological concept of capital has great promise for addressing several facets of contemporary consumer culture, as shown by Holt’s (1998) research in the United States and Tapp and Warren’s (2010) study in Europe.

Both the strengths-based (capabilities) approach (e.g., Sen 1997) and the deficits-based (resources) approach (e.g., Baker et al., 2005) to human capital neglect the residual resources generated from traumas and the benefits that result from them. This residual feature is central to the concept of coping capital that we propose based on Bourdieu’s basic formulation of cultural capital. To further our contribution, we recognize two types of coping capital: intentionally acquired and unintentionally acquired, the benefits of which may be determined using either a forward-looking (prospective) or a backward-looking (retrospective) approach. Furthermore, benefits might be anticipated or unanticipated in intentional coping with stresses, but benefits are predominantly unanticipated in unintentional coping activities.

By conceptualizing coping capital, we make a domain-level conceptual advance in the research area of consumer coping. Conceptual advances at the domain level are critical to the field’s development as they help identify “new and unexplored areas of study” (MacInnis, 2011, p. 142). Furthermore, our paper ‘functionally’ exemplifies “theory adaptation” by seeking to revise our understanding of consumer coping (viewed as a domain theory) by drawing on Bourdieu’s conceptualization of capital (viewed as a method theory—see Jaakkola 2020). The method theory’s role is generally “to provide some new insight into the domain theory—for example, to expand, organize, or offer a new or alternative explanation of concepts and relationships” (Jaakkola 2020, p. 20; see also Lukka & Vinnari, 2014).

We structure our conceptual article as follows. We begin with a review of the consumer coping literature, a concise note on strengths (capabilities) versus deficits (resources) approaches, and a discussion of Bourdieu’s notion of capital and previous conceptualizations of new forms of capital. We then introduce our conceptualization of coping capital, followed by some competing frameworks that we consider relevant to our conceptualization. Before offering our discussion section where we propose some future areas of research, we provide two short case studies illustrating the potential application of coping capital acquisition. One is on consumers’ pursuit of painful experiences possibly leading to the acquisition of *intentional* coping capital, and the other is on the Black oral tradition of ritualized insults conceivably generating *unintentional* coping capital. Before concluding, we suggest some theoretical questions for future research.

## Literature review

Lazarus and Folkman’s (1984) formulation of coping as “*constantly changing cognitive and behavioral efforts to manage specific external and/or internal demands that are appraised as taxing or exceeding the resources of the person*” (p. 141, original italics) is the most frequently cited definition of coping. Based on this definition, several scholars have polarized the coping phenomenon into the mutually exclusive aspects of managing problems and managing emotions (Duhachek, 2005). Problematizing this dichotomy, Bandura clarified that “successful coping usually requires both problem solving and stress management” and emphasized our strategic assessment of our coping self-efficacy is something that we use to address our life’s trials (Bandura, 1997, p. 149). He claimed that a person with high perceived self-efficacy might keep working despite persistent adverse outcomes for more extended periods and, therefore, better cope with them. “Perceived self-efficacy is concerned with judgments of how well one can execute courses of action required to deal with prospective situations” (Bandura, 1982, p. 122). In addition to problem-focused and emotion-focused coping, there are several other important coping groups and divisions including engagement (or approach coping) and disengagement (or avoidance coping); accommodative and meaning-focused coping; and proactive coping (Carver & Connor-Smith, 2010).

In our conceptualization we endeavor to offer an alternative explanation for understanding consumer coping by drawing on Bourdieu’s sociological notion of capital. This section is divided into three parts. First, we review the consumer coping literature to emphasize the infrequent attention given to the idea of consumers’ coping-related growth. Next, we present a brief note on the strength-based versus deficit-based approach. Finally, we offer a brief discussion of Bourdieu’s sociological view of capital that underpins our conceptualization of coping capital. In this third part on Bourdieu’s capital, we also provide a brief description of previously conceptualized new forms of capital.

### Consumer coping

Prior marketing and consumer studies have examined the phenomenon of coping from diverse perspectives. A large portion of the existing research on consumer coping has focused on coping strategies of shoppers in general (e.g., Trocchia, 2004; Whiting, 2009) and specifically those used by disadvantaged subpopulations. Studies on the coping strategies of functionally illiterate consumers (Viswanathan et al., 2005; Wallendorf, 2001), innumerate consumers (Viswanathan & Harris, 2001), visually impaired consumers’ coping with marketplace engagements (Balabanis et al. 2012; Falchetti et al., 2016), coping strategies of those with auditory disabilities vulnerable to sensory overload present in servicescapes (Beudart et al., 2017), low-educated consumers’ coping with stressful purchase encounters (Adkins & Ozanne, 2005), and coping strategies of consumers with English as a second language (ESL) (Viswanathan et al., 2010) are examples of studies of disadvantaged subpopulations. Other consumer coping studies have examined the stress of attempting to master technological innovations (Mick & Fournier, 1998); stressful decision-making in new technology product adoption (Cui et al., 2009); coping among consumer activists committed to eating local food (Bingen et al., 2011) and with food risk concern (Yeung & Yee, 2012); rumination and postconsumption guilt (Saintives & Lunardo, 2016); ethnic differences in coping (Ong & Moschis, 2009); and consumers’ coping with appliance failures (Donoghue & Klerk, 2012) and service failures (Zourrig et al., 2014; Tsarenko & Strizhakova, 2013; Gelbrich, 2010).

Marketing scholars have also looked beyond coping strategies to see what role consumers’ resources play in developing coping strategies. For instance, Yurdakul & Atik (2015) demonstrate low-income consumers’ use of political ideologies and religious beliefs to develop coping strategies and respond to poverty. Their study illustrates “the empowering aspect of religious arguments in providing low-income consumers with the strength to cope by resisting consumer culture and re-creating meaning beyond consumption” (p. 331). Viswanathan et al. (2021) focus on the role of marketplace literacy education on low-income and low-literate consumers’ coping with systemic economic macro shocks like India’s demonetization. They define the human capital resource of marketplace literacy education as “skills and knowledge, self-confidence, and awareness of rights as customers and entrepreneurs who are often small retailers” (p. 181).

Some other broader issues of coping have also been addressed in consumer research. Examples include marketplace discrimination faced by African-American men (Crockett, Grier and Willaims (Crockett et al., 2003), facing social disapproval and negative stereotypes from poverty (Hamilton & Hassan, 2010), coping with the tensions associated with practicing anti-consumption of alcohol in a setting where the prevailing norms are based around excessive alcohol consumption (Piacentini & Banister, 2009), coping with consumption stressors caused by natural disasters (Baker & Baker, 2016; Sneath et al., 2009), homeless children’s coping with material losses through fantasies of being rich and living in future homes with their cherished possessions (Hill, 1992), and overweight consumers’ coping with ambiguous messages that attract them to an insalubrious diet and simultaneously challenge them with images of a slender body (James et al., 2011). A significant contribution to coping scholarship was made by Pavia and Mason’s (2004) ethnographic study in the context of consumers suffering from terminal illnesses. Their study confirms the importance of the issues involved in determining coping strategies, supports the ‘reflexive’ nature of coping and establishes the significance of consumption as a coping mechanism across the different stages in the coping process.

While these studies have considerably informed our understanding of the coping phenomenon, the “consumer coping literature has focused predominantly on the immediate and short-term psychological consequences of stress” (Duhachek, 2008, p. 1072). Furthermore, few of them have considered consumer coping as a source of positive benefits rather than a compensatory mechanism or a matter of necessity. In considering consumer financial stress, Henry (2004) aptly noted that we may fail to appreciate that “stressful circumstances often hold positive potential for some form of growth” (p. 379). Similarly, in the discipline of sociology, Gutiérrez (1994) advocates for a shift in coping research by pursuing an empowerment approach, which would enable us to understand how stress responses can lead to positive change. Evidence exists that coping resources such as ‘resilience’ “transfer and rotate to foster empowerment in economically vulnerable groups” (Hutton, 2016, p. 267). The *unintended* benefits from coping with stressors may potentially carry over to dealing with hardships faced in other contexts of our lives and possibly much later in our lives. Consumer coping strategies, for example, can contribute to the development of a positive self-identity, which has significant implications for consumer empowerment and agency (Hamilton & Catterall, 2008). Recently, Kapoor & Belk (2022) emphasized the importance of the coping process over a person’s lifetime and demonstrated coping-related growth in gay men’s choices of altruistic careers. This suggests that we can observe our coping behavior’s productive effects in various contexts in our later lives. Building on this line of argument focusing on the growth-related aspects and the potential positive by-products of coping, we attempt to conceptualize coping capital mobilization by applying Bourdieu’s sociological concept of capital. In the next section, we will consider strength-based and deficit-based approaches, which, take little notice of the additional resources and benefits that may be derived from coping with adversity.

### Strength-based vs. deficit-based approach

In our endeavor to conceptualize coping capital, it seems essential to discuss the strength-based approach (focused on human capabilities) and deficit-based approach (focused on existing resources). This argument picks on two streams of thought—Amartya Sen’s work on capabilities and Baker et al.'s (2005) discussion on consumer vulnerability. Sen (1997) distinguishes between the perspectives of *human capital* and *human capability* while claiming that both place humanity at the forefront. According to Sen, human capital centers on our agency to expand production possibilities through our skills, energies, and knowledge, while human capability concentrates on “the ability of human beings to lead lives they have reason to value and to enhance the substantive choices they have” (Sen, 1997, p. 1959). The capability approach consists of two cardinal concepts: functionings and capabilities. The term ‘functionings’ refers to “beings and doings,” and it encompasses everything from being nourished and having good health to more complex accomplishments like happiness and self-respect (Sen, 1992, p. 39). ‘Capabilities’ is a closely related concept that refers to varying configurations of functionings indicating “the person’s freedom to live one form of life or another” that directly affect the person’s well-being (ibid., p. 40).

Sen’s strength-based view argues that we must expand our horizons by considering (expansion of) human capabilities in addition to (accumulation of) human capital. The Capability Approach (CA) focuses on a person’s capability to choose from a variety of practical alternatives available to them and to live the kinds of lives they have reason to value (Sen, 1997). To put it another way, Sen’s CA framework focuses on what people can do with their available means and resources, i.e., how to turn resources into meaningful “functionings” or states of “being and doing,” such as being happy and fulfilled. Sen’s framework emphasizes a person’s agency and freedom in reflectively choosing from their available resources. As we shall see, this aspect of agency is more fitting in the case of ‘intentional coping capital.’ It may, however, be irrelevant or just tangentially present in ‘unintentional coping capital,’ whose identification, we argue, is predicated on the realization of the benefits accrued from the reservoir of resources acquired and/or accumulated unintentionally.

An alternative view focuses on people’s deficits, some of which are innate, others caused by external factors, and still others caused by a combination of the two (Baker et al., 2005). Some examples of internal factors are a person’s self-concept and motivation states as opposed to external systemic factors and societal problems of discrimination and unequal resource distribution. A recent study with adolescents that illustrates how conditions of vulnerability may be either imposed (for example, by social institutions) or deliberate (for example, when someone decides to engage in hazardous activities) is notable in this context (Badat & Tanner, 2021). Under such circumstances, “[c]onsumers are required to call upon the resources at their disposal to work through the situations with which they are presented in their daily lives” (Baker et al., 2005, p. 6). For the most part, the deficits approach accentuates setbacks, inadequacies, deficiencies, incapacitation, sources of risks, and excessive reliance on external resources, besides suggesting that coping is a way of managing a finite set of resources. As indicated previously, we emphasize the aspects of the additional resources generated from coping with hardships and the benefits that result from these resources. In the next section, we review Bourdieu’s formulation of capital.

### Bourdieu’s concept of capital

The concept of capital (and its various forms) is one of Bourdieu’s central theoretical concepts, among others, such as habitus, field, and practice. According to Bourdieu (1986), capital is defined as “accumulated labor…which, when appropriated on a private, i.e., exclusive, basis by agents or groups of agents, enables them to appropriate social energy in the form of reified or living labor” (p. 241). This labor disrupts the distinction between the material and the symbolic. The notion of capital is not limited to money and property but extends to the social, symbolic and cultural. Bourdieu placed such a heavy emphasis on the concept of capital that he went on to argue that accounting “for the structure and functioning of the social world unless one reintroduces capital in all its forms and not solely in the one form recognized by economic theory” was impossible (Bourdieu, 1986, p. 242). Bourdieu contended that capital accumulated over time can generate profits and reproduce itself in various ways. He identified four forms of capital namely economic, cultural (embodied, objectified, and institutionalized), social, and symbolic.

According to Bourdieu, economic capital is the first form of capital and sums up the financial resources that people try to accumulate. If they are not yet monetized, the person can subsequently convert economic resources to money and standardize them in the form of property rights. Such economic capital can be used to gain even more capital. The second form of capital, cultural capital exists in three states. In the embodied state it can exist as enduring dispositions of the mind and the body; in the objectified state as cultural goods such as books, images, instruments, etc.; and in the institutionalized state as educational qualifications. Cultural capital is much more than materialistic economic capital. It also refers to the knowledge of cultural codes and of conduct, behavior and discussion. The third form of capital, social capital, refers to the institutionalized relationships and group memberships that enable members to gain access to joint capital as well as call upon each other. The possession of social capital may yield material and non-material gains. The fourth form of capital, symbolic capital, is linked to recognition and prestige, and is mainly merit-oriented, referring to the power of a person in society. Symbolic capital can be “readily convertible back into economic capital” (Bourdieu, 1977, p. 179). The three previous forms of capital culminate in and comprise symbolic capital.

From the Bourdieusian perspective, capital has some central characteristics. First, it is relational. The relational nature of capital posits that “*capital does not exist and function except in relation to a field*” (Bourdieu & Wacquant, 1992, p. 101, original italics). In the social space, which is organized into various fields, there is a struggle for power. While Bourdieu discusses a variety of fields, including scientific, educational, intellectual, cultural, and religious, his primary focus has been on economic and political fields, as well as the blend of the two that he designates the ‘field of power’ (Hesmondhalgh, 2006, p. 212). The field of power is “the space of the relations of force between the different kinds of capital, or more precisely, between the agents who possess a sufficient amount of one of the different kinds of capital to be in a position to dominate the corresponding field” (Bourdieu, 1998, p. 34). Furthermore, an actor’s status in the field is determined by the amount of capital they have relative to the other field participants. Second, capital is accumulative. As previously stated, Bourdieu (1986) described capital as “accumulated labor” that takes time to generate. And, third, capital transubstantiates. That is, it reproduces and translates into other different capital forms.

Despite critiques of Bourdieu’s social theory being “deterministic and circular,” with objective structures producing habitus, habitus determining practice, and practice, in turn, reproducing structures (Jenkins, 1982, p. 270), some scholars make a compelling case for the applicability of Bourdieu’s theory in today’s world. For example, Bourdieu’s theory, according to Holt (1998), is a series of “sensitizing propositions” concerning the interconnections between tastes, fields, social conditions, and reproduction, rather than a “nomothetic theory” based on law-like generalizations (p. 6). Unsurprisingly, numerous scholars have expanded on Bourdieu’s concepts. The concept of capital, for example, has been extended to explain various phenomena through a series of formulations, many of which refer to Bourdieu. Scholars have conceptualized new forms of capital, such as spiritual capital (Verter, 2003), psychological capital (Ortner, 2002), erotic capital (Hakim, 2011), masculine capital (Vasquez del Aguila, 2014), queer capital (Kjaran & Jóhannesson, 2016), ageing capital (Simpson, 2014), cognitive capital (Henry, 2004), and emotional capital (Cottingham, 2016; O’Brien, 2008). Table 1 below covers the definitions of each of these new capital forms.

**Table 1:** Conceptualization of new forms of capital

Capital form	Author & Publication details	Description
Spiritual Capital	Verter (2003). Spiritual capital: theorizing religion with Bourdieu against Bourdieu. *Sociological Theory*, 21(2), 150–174.	Embodied state: Spiritual capital encompasses “the knowledge, abilities, tastes, and credentials an individual has amassed in the field of religion, and is the outcome of explicit education or unconscious processes of socialization” (p. 159) Objectified state: Spiritual capital “takes the form of material and symbolic commodities- votive objects, exegetical texts, and ritual vestments, as well as theologies, ideologies, and theodicies” (p. 159). Institutionalized state: Spiritual capital refers to “the power that churches, seminaries, and other religious organizations exercise to legitimate an arbitrary array of religious goods, promote the demand for these goods, and feed the supply by bestowing qualifications on a select group of authorized producers” (p. 160)
Psychological Capital	Ortner (2002). Subjects and capital: a fragment of a documentary ethnography. *Ethnos*, 67(1), 9–32.	Psychological capital refers to “the quality of love and support that one gets from one’s important social relationships, and particularly—for the years of growing up—from one’s immediate family” (pp. 20–21)
Cognitive Capital	Henry (2004). Hope, hopelessness, and coping: a framework for class-distinctive cognitive capital. *Psychology & Marketing*, 21 (5), 375–403.	Cognitive capital refers to the idea that “one’s mix of psychological dispositions operates as a system that can be thought of as an important type of resource that is gradationally distributed with social class level” (p. 375)
Erotic Capital	Hakim (2011). *Honey money: The power of erotic capital*. Allen Lane: London.	Erotic capital entails charm, sociability and sexual expertise that can all be used to advance in careers and other areas of life.
Masculine Capital	Vasquez del Aguila (Vasquez 2014). Masculine capital, homophobia and homoeroticism. In A. Amodeo and P. Valerio (Eds.), *Hermes: Linking Networks to Fight Sexual Gender Stigma*. Naples: Liguori Editore.	Masculine capital is “a form of cultural capital that provides men with the necessary ‘masculine’ skills and cultural competence to achieve legitimacy and social recognition as respected men” (p. 10)
Emotional Capital	Cottingham (2016). Theorizing emotional capital. *Theory and Society*, 45(5), 451–470. O’Brien (2008). Gendered capital: Emotional capital and mothers’ care work in education. *British Journal of Sociology of Education*, 29(2), 137–148.	Emotional capital refers to “*one’s trans-situational, emotion-based knowledge, emotion management skills, and feeling capacities*, which are both socially emergent and critical to the maintenance of power” (p. 454, original emphasis). Emotional Capital is “a resource that specifically involves mothers in performing what they identify as the ‘moral work’ of being a good mother through caring for their children, including educational care” (see O’Brien, 2008, p. 138)
Queer Capital	Kjaran, J. I., & Jóhannesson, I. Á. (Kjaran & Jóhannesson 2016). Masculinity strategies of young queer men as queer capital. *NORMA*, 11(1), 52–65.	Queer capital refers to “ideas, looks, and behaviors that can be adopted as social strategies striving to gain symbolic value within the field of masculinity” (p. 54)
Ageing Capital	Simpson (2014). Differentiating selves: middle-aged gay men in Manchester’s less visible ‘homospaces’. *The British Journal of Sociology*, 65(1), 150–169.	Ageing capital refers to “the gains in self-esteem through ageing” (p. 151).

Despite the large corpus of coping literature that exists in consumer research and that we reviewed above, there has not been enough attention paid to growth-related elements of coping with adversity. The strengths-based and deficit-based approaches also take little notice of the additional resources and benefits derived from overcoming adversity. We bridge these gaps by drawing on Bourdieu’s concept of capital.

## Conceptualizing coping capital

**Fig. 1:**
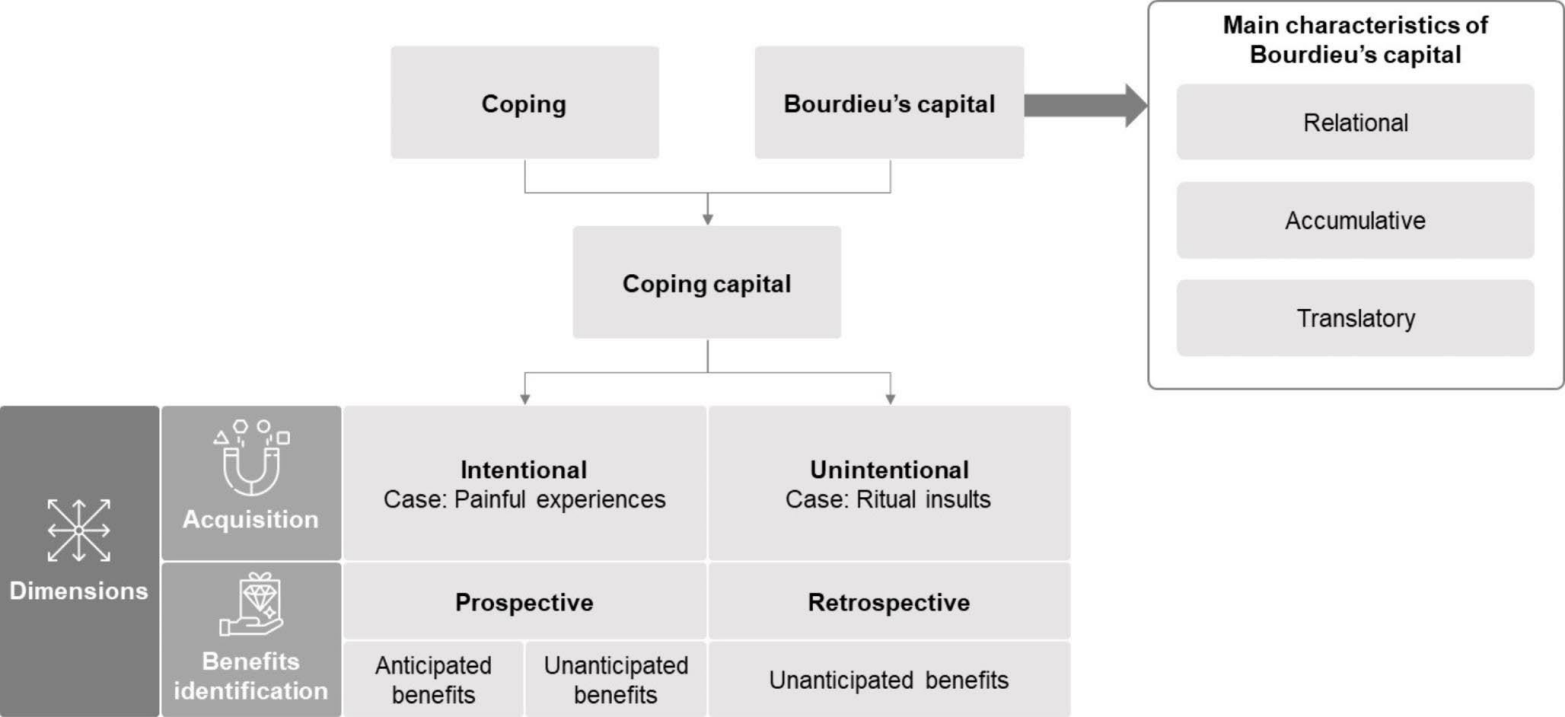
Framework of coping capital

We define coping capital as the intentional or unintentional acquisition and/or accumulation of resources, such as emotional and epistemic-competencies and skills resulting from coping with adversity, that *may* thereafter exist in an embodied state in the form of mental and physical dispositions that later provide benefits in life. There are two main dimensions of coping capital: acquisition and benefits identification. Figure 1 illustrates these dimensions. These will be discussed in further detail in the following paragraphs.

In our literature review, we referred to a distinction between coping resources and coping strategies. The coping resources that Hutton (2016) referred to are the resources, such as income, education, and resilience, that we draw on to mitigate stress during unfavorable situations we face in our lives. From these subsequent realizations, it seems, we discover that we have the needed resources available already. We do not conflate coping capital with *existing* coping resources. Nonetheless, coping capital may lead to the generation or accumulation of additional resources such as hardiness and resilience. According to our conceptualization, coping capital is a resource, but one that emerges from adversity and that leads to benefits. As such, coping capital is also distinct from the other life skills (e.g., communication skills, problem-solving skills, self-awareness) that we develop as part of our normal development. It is more applied (e.g., a need to escape from a predator) rather than tangible resources (e.g., income) and hypothetical ones (e.g., education).

We argue that the characteristics of Bourdieu’s capital (relational, accumulative, and translatory) can be applied to the notion of coping capital we propose. The possession of an actor’s coping capital relative to other field-actors would determine their position in the field of power through the different competencies they possess because of the hardships they confronted. That is, those rich in coping capital will have an advantageous position over those that do not. Similar to those possessing masculine capital benefitting from achieving “legitimacy” and “social recognition” (Vasquez del Aguila, 2014, p. 10), and those with ageing capital benefitting from enhanced “self-acceptance” and “capacity to resist cultural pressures” (Simpson, 2014, p. 153), those with coping capital may reap many unanticipated benefits. In a subsequent section we describe some of the potential benefits of coping with hardship in our two case studies of consumers’ pursuit of painful experiences and the Black oral tradition of ritualized insults.

Coping capital is also accumulated over time and is sometimes generated through the unintentional expenditure of labor. The intentional expenditure of labor is shown by consumers’ pursuit of painful experiences such as Tough Mudders that we discuss later as a part of a case study we offer, but the unintentional expenditure of labor is exemplified by people’s coping with obstacles that they do not purposefully choose. The concept of coping capital that we are proposing has some similarities with Bourdieu’s habitus and embodied cultural capital in that the former may also be seen as a form of long-lasting dispositions of the mind (see Bourdieu 1986, p. 243). Bourdieu’s notion of embodied cultural capital “presupposes a process of embodiment, incorporation, which, insofar as it implies a labor of inculcation and assimilation, costs time, time which must be invested personally by the investor” (Bourdieu, 1986, p. 244). In Bourdieu’s case this is often intentional such as taking a class in a useful subject. In the case of coping with adversities, the costs may also be emotional and psychic resulting from stress and decreased life quality (Hewlett, 2011; Prothro & Smith, 1995). Habitus is ingrained in “family upbringing (socialization within the family) and conditioned by one’s position in the social structure” (Edgerton & Roberts, 2014, p. 195). As Edgerton and Roberts point out, Bourdieu used the term ‘dispositions’ to describe both embodied cultural capital and habitus; nonetheless, all dispositions do not qualify as capital unless they generate benefits to the individual.

As with other forms of capital, coping capital may also reproduce or translate into other forms of capital. For instance, learning to calm anxiety may be difficult, but once it is mastered, we may help others to do the same and thereby gain monetary, social, or status rewards. Referring to Bourdieu’s notion that “capital finds its way to capital” (Bourdieu, 1998, p. 19), unless we formulate coping capital, we will not be able to show how early life experiences reshape other forms of capital in different contexts in our lives. That is, one of the most compelling reasons for conceptualizing coping capital, we believe, is its potential contribution to our understanding of how coping capital transubstantiates into other forms of capital. We suggest that adverse experiences may provide a positive influence and another kind of capital that we call coping capital. In boxing, this is called learning to take a punch.

There are two dimensions of coping capital—acquisition and benefits identification—that we mentioned above (see also Fig. 1). We underline the aspects of ‘intentionality’ and ‘unintentionality’ in defining coping capital because although coping is conscious and intentional stress adaptation, the resource or skills generating from it and the benefits resulting from it can often be unintended and temporarily unfolding. Nonetheless, as we will see, some instances of consumers’ conscious pursuit of painful experiences necessitate consideration of coping capital derived from even our intentional acts to obtain them. Therefore, we classify coping capital into those that are intentionally acquired and those that are unintendedly acquired. Furthermore, the dimension of benefits identification is important in appreciating the concept of coping capital.

The appropriation of social energy, according to Bourdieu, is a mechanism that renders “accumulated labor” as capital. On the other hand, Polanyi (1944/2001) regards land, labor, and money as “fictitious commodities” that resulted from the broad commodification that accompanied the Industrial Revolution. Both labor and coping existed long before the Industrial Revolution. However labor capital may not have been recognized as such until it became paid labor whereas coping capital may not have been imaginable as such until the concomitant identification of “to cope with” as a figurative form meaning “To contend with, face, encounter (dangers, difficulties, etc.). Often implying successful encounter” (O.E.D. 2000). By looking forward or using a prospective stance, a person might uncover rewards such as a feeling of success and status appreciation in consumers’ purposeful actions of coping with physical discomforts, such as in mountaineering. The benefits here might be anticipated or unanticipated. Another case in point is the example of schooling conflicts faced by low-income African American youth hailing from a history of slavery and discrimination that we describe below.

In contrast to Bourdieu’s “dominant cultural capital” as high-status cultural codes, Carter (2003) refers to “non-dominant cultural capital” as “a set of tastes, or schemes of appreciation and understandings, accorded to a lower-status group, that include preferences for particular linguistic, musical, or interactional styles” (p. 138). As Carter suggests, to cope with the socio-economic challenges associated with fitting in with the ‘cultural default’ of the white, middle-class tastes of dress and expression in school environments, some ethnic minority students juggle between using both this dominant cultural capital (in school settings) and non-dominant cultural capital (within their communities). This aspect is also called subcultural capital (Thornton, 1995). Carter further states that those “who choose the balancing act of maintaining both dominant and non-dominant cultural capital *are likely to* acquire valued status positions within both their status community and the wider society” (p. 139, emphasis added). While the ongoing negotiations of students and the cost-benefit analysis of the social condition with which they want to cope and behave properly in is “intentional” and a deliberate effort, the benefits accruing from it, for example, Carter’s mention of the “likelihood” of their acquisition of valued status positions in the broader society, i.e., outside the immediate context for which the technique was used, maybe unanticipated. Such unintentional benefits are an important aspect of coping capital.

Similar to the example of a dialectic tension between standing out and fitting in discussed above in the case of the African American school goers is an alternative status system and a key driver in consumer culture—that of being cool. “Cool is a particular impression-related verbalized and embodied performance” (Belk et al., 2010, p. 184). The authors refer to the status that coolness conveys as “cool capital.” To gain cool capital a person not only needs to stand out and be different, but also be indifferent to the opinions of the peer group to whom one is cool (p. 201). Apparently, as the authors note, mostly minority people on the fringes of society, such as gangsters, delinquents, rappers, and sexual minority groups have been the originators of cool, which unintendedly became “a way of substituting a particular cultural capital for economic capital” (p. 189).

However, gains arising from the unintended acquisition of coping capital may be found reflexively by looking back and taking a retrospective stock of the difficulty experienced and coping with it. In this case, coping capital is mainly benefits-driven where even the identification of coping capital is contingent on the benefits that arise from it. Once we realize the advantages or benefits, we reap, we reflexively look back to see how coping with adversity generated resources leading to these benefits. That is, we discover the existence of coping capital through a retrospective approach. Also, identifying coping capital is contingent on realizing the unanticipated benefits that arise from it. For example, someone who accidentally wades into a deep spot in water that is suddenly over their head may learn to tread water and to turn the terror of anticipated drowning into the calm of learning deep water survival. The person may not appreciate until years later when they again unexpectedly encounter deep water that they have acquired the coping to calm themselves and deal with the situation. There may be several similar events more directly related to consumer behavior. Someone who has been scammed previously, for example, may know how to get their money back. Someone may have already had their credit card information stolen and is familiar with the protocol for contacting their bank or credit card company. Someone who has been in an auto accident understands what is required for insurance claims, someone who has driven on ice previously knows how to keep control of their vehicle when it skids, and someone who has attended auditions before knows what to anticipate. In such cases, the research methods we deploy may play a major role in helping participants to identify the existence of coping capital. Longitudinal studies or oral history methods may perhaps be suitable for this purpose.

## Competing frameworks

Jaakkola (2020) reminds us that besides “justifying why a particular extension or change of focus is needed, a theory adaptation paper must also show that the selected method or theory is the best available option” (p. 23). In this section, we seek to show that our choice of Bourdieu’s concept of capital is appropriate for our conceptualization of coping capital, which may be applicable in various contexts. We outline our assessment of some of the prominent theoretical frameworks in our consideration set that served as competing frameworks. These are cognitive capital theory (Henry, 2004), the theory of resilience, the theory of emotional capital, and the conservation of resources (COR theory). These theories and their points of similarities with and differences from our conceptualization of coping capital are discussed below.

Cognitive capital refers to the idea that “one’s mix of psychological dispositions operates as a system that can be thought of as an important type of resource that is gradationally distributed with social class level” (Henry, 2004, p. 375). Both cognitive capital and coping capital have the common aspects of coping with stressors and the generation of resources. The premise upon which Henry (2004) grounds his concept of cognitive capital is the systematic variation in psychological dispositions with social class where class-related resources are family/peer socialization, educational experiences, material resources, etc. “Those with more limited material resources will face greater adversity in terms of day-to-day stresses” (Henry, 2004, p. 392) that would in turn lead to hopelessness and maladaptive coping. However, while low social class may be one of the causes of stress, the foundational lever of coping capital is not social-class. The adversities that generated the coping capital and its subsequent uses are the phenomena of interest in our concept of coping capital.

A note on resilience is essential here, as there is a possibility that the readers may confuse the notion of coping capital with resilience. Generally, the term ‘resilience’ refers to “the process of, capacity for, or outcome of successful adaptation despite challenging or threatening circumstances” (Masten et al., 1990, p. 426). It is the ability to “maintain an unwavering belief that one will find one’s way out of troubles [and] surmount adversity and meet challenges” (Saleebey, 1996, p. 298). However, it is important to note that there are several definitions of resilience, and “[o]ne of the primary challenges for resilience researchers has been achieving consensus on definition” (see Walker, 2001, p. 292). For instance, some define resilience as “a return to the pre-adversity level of functioning, a return that can be either rapid or more gradual” (see Carver, 1998, p. 246). Carver (1998) further suggests that “the term resilience be reserved to denote homeostatic return to a prior condition” (p. 247). This view of resilience is more akin to the concept of recovery. Manyena et al., (2011) problematize this issue of bouncing back and instead argue that resilience should be viewed as a person’s ability to “bounce forward” following a disaster. It must be noted that both coping and resilience change “developmentally and experientially across one’s lifespan” (see Rice & Liu, 2016, p. 329). Resilience, according to Carver (1998), is a return to a pre-adversity level. There is no pre-adversity dimension in our conceptualization of coping capital. That is, there is no “homeostatic return to a prior condition” (Carver, 1998, p. 247) that could bring our study within the ambit of resilience. Nor is there an inherent bounce forward as there may be in some directed recovery efforts (e.g., Baker & Baker, 2016; Mannakkara & Wilkinson, 2013). Moreover, resilience is defined as “an inner resource used to cope with difficult experiences” in the consumer studies and psychological health literature (Hutton, 2016, p. 258). So, for Hutton, resilience is a coping resource that we draw upon and that is a stress mediator. Coping capital, as we conceptualize it, might involve resilience in the sense that coping with challenges may make people more resilient. But again, this is incidental rather than purposeful foresight.

Emotional capital, from a feminist perspective, is “a resource that specifically involves mothers in performing what they identify as the ‘moral work’ of being a good mother through caring for their children, including educational care” (see O’Brien, 2008, p. 138). Similar to our conceptualization of coping capital, emotional capital also serves as a resource that leads to future benefits. However, emotional capital has been referred to as “a gendered capital” (see O’Brien, 2008, p. 139) where notions of women as “natural carer[s]” and their “rejection of earthly and sexual pleasures” (p. 138) has been embraced. Such an aspect of natural inheritance may not necessarily be present in case of coping capital. In fact, the adverse contexts we live and cope with may or may not lead to the generation of such resources. Emotional capital and coping capital have certain similarities in terms of both being a resource and involving gains that arise with it. However, emotional capital, as described above, is not defined as something that develops from coping with hardship, which is central to our conceptualization.

Conservation of Resources (COR) is a general motivational theory of stress and resource utilization. According to the COR theory, “individuals strive to obtain and maintain that which they value—these things being termed ‘resources’” (Hobfoll & Freedy, 1993, p. 117). There are some principles underpinning the COR theory. First, in people’s striving for protection from loss of resource, “loss is more salient than gain” (ibid.). Second, although prevention of resource loss is more important than resource gain, gaining resources is consequential as they decrease the chance of loss and augment social status. Third, resource loss is compensated by other resources. Furthermore, the COR theory posits that “when loss occurs people engage in coping more actively in order to reduce the effects of loss” (ibid., p. 121). The COR theory is highly proactive as it suggests that people consciously “seek resource gains in order to avoid the possibility or severe consequences of future loss” (Hobfoll & Freedy, 1993, p. 123). Our formulation of coping capital is about resource accumulation resulting in benefits, rather than exclusively deliberate, compensatory, and proactive resource gain, as suggested by the COR theory. COR theory, for example, does not account for unintended resource acquisition/accumulation and the benefits that arise from it, which the coping capital notion does.

The unique aspect about our coping capital concept versus all of these rival theories is that it entails turning what is usually a negative life experience into a positive asset. Rather than each of the alternative theories having something to add to our formulation, they all stop short of anticipating this unique feature of coping capital. That is, while they all share a focus either on acquiring valuable resources or bouncing back from adversity, none of them envision a conversion of negative experience into positive formulation, as we show in our conceptualization of coping capital.

While we have attempted to justify our choice of Bourdieu’s notion of capital to conceptualize coping capital by presenting some competing frameworks here, it is also important to demonstrate the potential usefulness and applicability of the concept in understanding various other contexts (Belk & Sobh, 2019). A further emphasis is placed on the use of illustrative cases and anecdotes to “contextualize, exemplify and motivate conceptual contributions” (Vargo & Koskela-Huotari, 2020, p. 3). As such, we now turn to two illustrative cases as potential examples of acquisition of coping capital. One on consumers’ pursuit of painful experiences is used to demonstrate potential acquisition of intentional coping capital. The other is an unrealized example from anthropological literature about ritual insults in African American communities, which demonstrates how unintended coping capital may be acquired.

## Acquisition of coping capital: Potential examples

### Intentional coping capital: The case of painful experiences

Many consumers seek out painful experiences on purpose. The pursuit of purposefully experiencing pain is crucial to and inextricably linked to the “process of identity construction and making meaning of one’s identity and place in society” (Tung, 2020, p. 253). In the physical sense, pain is considered as “a subjective measure of a negative sensory experience of physical discomfort or damage” (Cutler et al., 2014, p. 154). However, pain can also be non-physical or psychological such as separation from a significant other (Bakan, 1968). Painful experiences can be encountered in many ways such as in extreme sports (e.g., steep mountain climbing), intense tourist experiences, and several religious rituals. The ethnographic study of the Tough Mudder adventure challenge by Scott et al., (2017) is a ground-breaking study that shows how painful experiences help participants redirect their attention on their body’s corporeality and craft their life narrative, while also uncovering “a new dimension of their humanity through their body” (p. 37). Dangerous activities, such as mountaineering, have tremendous “self-signaling” value since they allow a person to learn their true self—both their strengths and shortcomings (Loewenstein, 1999). Furthermore, as Loewenstein points out, climbing is a prestigious pursuit in certain coteries and, therefore, may serve as a means to generate symbolic capital in Bourdieu’s terms. In fact, sharing how you coped with painful events may also elicit empathy and social support from others, resulting in the accumulation of social resources (Leknes & Bastian, 2014)—or, in Bourdieu’s terminology, social capital. People get various advantages from participating in high-risk sports, including feelings of freedom, self-confidence, and self-efficacy (Celci, 1992).

The intentional pursuit of painful experiences may also lead to a buildup of coping capital. For example, research suggests that participating in extreme sports (such as kayaking and windsurfing) wherein death is a strong likelihood leads to a variety of “permanent, instant and unexpected” positive changes, involving the development of courage, humility, and open-mindedness toward the natural world in the participants (Brymer, 2009, p. 49). Sexual masochism, which is defined by a person’s intense desire to be exposed to painful experiences in sex play, is another example of intentional seeking of painful experience. Surprisingly, masochists who engage in such purposefully painful sexual encounters have been shown to have above-average coping skills and demonstrate “a strong relationship orientation” (Baumeister, 1997, p. 140). Even tattooing, which is considered a painful, but regulated kind of violence conducted on the body is shown to stimulate a body’s immune system possibly improving one’s long-term fitness (Piombino-Mascali & Krutak, 2020, p. 126). In fact, the hardening theory, which is “based on the belief that any physical and emotional stress exerted on young children will allow the individual to withstand strain, both physical and mental, in its later life,” is used to justify body modifications such as tattooing and scarification in many indigenous societies (Garve et al., 2017, p. 709). In the context of painful experiences such as tattooing, it is also argued that “self-violence and voluntary pain can be productive rather than destructive by enacting the virtue of self-detachment” (Cova, 2021, p. 66).

Painful experiences also clearly and abundantly permeate many faiths and religious rituals. Of interest to note is whether these self-inflicted painful and brutal ritual experiences provide any benefits to those who participate in them. Pain, according to a CCT study on pilgrimages, enables pilgrims to slow down and reflect on themselves, shifting their emphasis from the outward to the inner (Husemann & Eckhardt, 2019). Participants in contemporary Japanese firewalking consider that the practice has psychosomatic advantages and aids in mind-control and regulation (McClennon, 1988). Religious pain, according to Glucklich (2001), generates “states of consciousness” and “cognitive-emotional changes” that foster the individual’s link with God and impact their feelings of belonging to the wider community (p. 6). At both the micro-individual and macro-social levels, the benefits of such painful experiences can be observed. The psychotropic model of pain, for example, stresses pain’s analgesic characteristics, whereas the shared model of pain accentuates “bonding effects,” such as those observed in rites of passage when boys and girls are afflicted, as well as in rituals of mourning (Glucklich, 2001).

There are also several other examples of resource build-up occurring as a result of intentionally coping with stressful situations. For example, military training (ROTC) improves the decision-making and critical-thinking skills of those who participate (Kreft et al., 2014). In a similar vein, Outward Bound and daring experiences assist participants to build their leadership abilities and self-concept (Hattie et al., 1997).

### Unintentional coping capital: The case of ritual insults

There is evidence for the applicability of coping capital in the context of African Americans, in part through the socially-accepted folkloristic phenomena of ritualized insults and verbal jousting that serve to develop a “thick skin” (Bertucci & Boyer, 2013; Labov, 1972). We contend that the ritualized linguistic game ‘playing the dozens’ in such communities serves an example of coping capital that is later used in facing discrimination, prejudice, and subjugation. This Black verbal play (“playin’ the dozens”) involves a succession of interactive insults following a set of well-recognized rules that for some onlookers and participants is a mere vocal game providing them with some form of amusement and recreation (Dollard, 1939, p. 4). Participants engage in a verbal contest in an imaginary playfield wherein one participant attacks the other’s family member (Abrahams, 1962, p. 211). Ritual insult is a form of ritual duelling with words (Neu, 2008). The African Americans, for example, brace for insults and aggression from whites by trading verbal insults among their own community members (Lefever, 1981). Through such participation, they learn to rise above verbal abuse and prepare themselves to withstand the hostilities of the white community (Abrahams, 1962; Dollard, 1939). In contrast to ‘ritual insult’ that entails insulting the other’s family members (mostly mother), the ‘applied insult’ is a personal insult focused on the addressee himself, which is generally responded to by denying, excusing, or mitigating (Schwegler, 2007, p. 112).

Although different scholars have explained the Black oral tradition and cultural pattern differently, the phenomenon serves as an adaptive “way of coping” by which African Americans displace their aggression toward whites who demean them by verbally insulting their own community members (Abrahams, 1962; also see Neu, 2008, p. 62) as mocking openly at and displaying resentment against the white people would supposedly incite verbal punishment (Dollard, 1939, p. 21). This cultural pattern has expressive and instrumental value, fulfilling various social and psychological purposes for the participants (Lefever, 1981). For example, as Abrahams (1962) points out, the “dozens” serves several important functions beyond that which is apparent, such as they help the lower-class Negro youth, living in a matriarchal system, to develop his masculine identity and sexual power.

By insulting each other (often in the “Yo’ mother…” genre), African Americans (especially males) not only learn to deal with a hostile white society (Lefever, 1981) by partaking in verbal insults against each other but also reap other benefits, such as developing a “thick skin” to be able to endure and rise above verbal abuse. For example, such an engagement trains them in the particular gender relationships that exist in their community (Hannerz, 1969) and hones their linguistic ability to improve their status (Lefever, 1981). Dollard, Lefever, and Garner refer to other control functions associated with playing the dozens. These include developing the ability to restrict their attacks to the verbal mode (Dollard, 1939, p. 13), acting as a nonviolent way of social control (Lefever, 1981, p. 80), and serving as rhetorical techniques to moderate interpersonal conduct in other ordinary situations of conflict (Garner, 1983, p. 57). There is also some evidence that the “nonliteralness and innovative word connotations” heavily used in the Black language play enhances the participants’ comprehension of figurative language (Delain et al., 1985, p. 171). In the light of the above, it is likely seen as an opportunity to swap coping skills for other skills that will aid in education, career, and social relationships in general.

African American peoples’ oral tradition of ritual insults can be considered as a set of techniques to develop coping capital, which may translate into other forms of unintended life skill capital in the community members’ lives. The improvement of their language dexterity and verbal rather than physical responses to discrimination can be construed as building cultural capital, while the subsequent rise in their status in the eyes of peers builds their symbolic capital. This is also institutionalized in so-called “battle rap”—another competitive genre of verbal dexterity, in this case enacted though spontaneously producing rap lyrics to “big one’s self up” (Mavima, 2016). Battle rap is a more formal artist/DJ competition that codifies the “dozens” concept and relies on coping capital skills learned on the street. Eminem came up this way as a rap musician.

## Discussion

As mentioned in the literature review, few coping studies have considered coping as a source of positive benefits rather than a matter of necessity. That is, the importance of coping-related growth has yet to be sufficiently acknowledged and addressed. Furthermore, both the strength-based (capabilities) and deficit-based (resources) approaches that we discussed in our paper take little notice of the additional resources generated and benefits that result from them, which are central to the concept of coping capital that we propose using Bourdieu’s notion of capital. Therefore, this study sets out to assess how can coping with adversity generate resources that provide benefits to the individual—benefits that are transferable to various contexts of life. In other words, this study shows how can coping with difficulties be viewed as capital. To this end, we use Bourdieu’s sociological notion of capital to conceptualize coping capital. We elaborate on the main characteristics (relational, accumulative and translatory) of Bourdieu’s notion of capital to show how they apply to our concept of coping capital).

We define coping capital as the (intentional or unintentional) acquisition and/or accumulation of resources, resulting from coping with adversity, that later provide benefits in life. We identify two main dimensions of coping capital: acquisition and benefits identification. Accordingly, we recognize that coping capital can be both intentionally as well as unintentionally acquired. To bolster our argument, we offer two brief case scenarios. One case of consumers’ pursuit of painful experiences such as mountaineering and kayaking and partaking in painful religious rituals may generate intentional coping capital. The other case on “The insult ritual known as ‘playing the dozens’ … studied extensively for its impact on young African Americans” (Wooten, 2006, p. 189) is a potential case scenario that illustrates the acquisition of unintentional coping capital. The second important dimension of coping capital is that of benefits identification. We may identify benefits from intentional and unintentional coping capital by either looking forward and using a prospective stance or by looking backward and taking a retrospective stock of the difficulty experienced and coping with it.

We also evaluate several important theoretical frameworks in our consideration set that acted as competing frameworks as part of our conceptual effort. These include cognitive capital theory (Henry, 2004), resilience theory, emotional capital theory, and resource conservation theory (COR theory). Our conceptualization of coping capital, which makes a domain-level conceptual advance in the research area of consumer coping, can be applied to many other contexts. Our endeavor to integrate two well-known topics—consumer coping and Bourdieu’s cultural capital—in order to create the novel concept of coping capital involves “combinational creativity” (Stewart, 2020, p. 66), a technique that helps us look at the phenomenon of consumer coping in a new way. By linking work between marketing and consumer research on one hand and coping and sociology via Bourdieu on the other, we augment our understanding of the coping phenomenon. We hope that our idea of coping capital will contribute to the advancement of the domain of consumer coping and will help identify potentially promising agendas for future coping scholarship. We offer some such scenarios where our concept of coping capital may be useful.

### Phenomena that should be examined or reexamined in light of coping capital

The theoretical framework we offer can be used to better examine or reexamine a variety of consumer traumas and coping phenomena such as those involving psychological problems, physical and sexual abuse, pathological traumas, philosophical and existential dilemmas and more general accidents and natural disasters. Each of these phenomena potentially produces coping capital that can be helpful in dealing with future crises in the same or different contexts. The concept of coping capital we propose may be applicable, for instance, in disadvantaged people’s choices of careers in social work or community care where they can incorporate their life experiences into their future careers. There is evidence that most people who chose social work as a career have suffered psychological trauma or have grown up in dysfunctional families (Rompf & Royse, 1994). Refugees’ experiences of crime and violence often lead them to strive for altruistic vocations (Tlhabano & Schweitzer, 2007). And members of different sexual minorities may decide to become political activists and campaigners or work for LGBTQ + non-government organizations. In such circumstances, their coping capital may be traded for monetary capital in career choices or for social capital in responses to a post about earlier experiences, resulting in the #MeToo movement for various LGBTQ + persons. Perhaps previous life experiences have provided those in all of these cases a sense of purpose and direction, as well as a platform on which to construct their identities (Burrow & Hill, 2020).

Many people choose to become activists in social movements because of their backgrounds (see Valocchi, 2012; Hunt & Benford, 1994). For instance, Icelandic people with intellectual disabilities as well as social disadvantages leading to their educational, vocational, and social exclusion, contribute to the development of spaces of self-advocacy (Björnsdóttir & Jóhannesson, 2009). These choices are often sociohistorically shaped and may entail “embodied, holistic perceptual comprehension characterized by the experience of perfect fit” as explained by fits-like-a-glove (FLAG) theory (Allen, 2002, p. 520). Studies may also reexamine the accumulation and translation of coping capital in the context of subsistence marketplaces i.e., “consumer and entrepreneur communities living at a range of low-income levels” (see Viswanathan & Venugopal, 2015, p. 228). Hill (1992) and Hill & Stamey (1990), for example, found forward-looking coping benefits among homeless people, as well as backward-looking unintentional coping benefits acquired by learning where to scavenge.

In Adkins and Ozanne’s (2005) research on low-literate consumers’ marketplace interactions, some participants made amends for their poor literacy skills by “memorizing” and employing visual aids, while others constrained their shopping to a manageable familiar range that they found comfortable. Elsewhere, “context-based pictorial representations” were also found to be used by functionally illiterate consumers (Viswanathan et al., 2005, p. 21). It might be insightful to further investigate low-literate and functionally illiterate consumers’ use of coping through memorizing and pictographic thinking. We anticipate that these techniques might contribute to or derive from coping capital in other contexts such as in their professions. Rethinking research on homeless consumers’ coping techniques from the coping capital perspective proposed in this study might usher in new lines of discoveries. Existing studies show that homeless women cope with shelter life by building profound ties with the Roman Catholic nuns who run the shelters (Hill, 1991). Similarly, homeless children form deep ties with other children and volunteers (Hill, 1992). Quite possibly, in Bourdieu’s terms, the development of such strong relationships may generate coping capital (as well as social capital) arising from dealing with the difficulties of homelessness. Similar capital may also develop from sex workers’ coping strategies in a libidinal market, which include connecting with similar others who share their life circumstances (Mitra et al., 2022). Table 2 highlights consumer research studies that may be leveraged to investigate or reinvestigate the applicability of coping capital.

**Table 2:** Phenomena of consumer coping that can be examined for coping capital

Examples of coping with stressors	Related works in consumer research	Application of coping capital
Coping with literacy issues in marketplace engagements	Adkins & Ozanne (2005) Viswanathan et al., (2005) Viswanathan & Harris (2001)	Coping capital from illiterate consumers’ dealing with shopping cues can be used to deal with traffic signs, notices they receive in the mail, and television listings. These are translations primarily to economic capital (e.g., banking)
Coping with homelessness	Hill (1991) Hill (1992) Hill & Stamey (1990)	The street skills learned by the homeless out of desperation and need for survival may translate into social capital by being able to help new homeless people also survive and thus building social capital among those they help.
Coping with stigmatization	Mitra et al., (2022)	Sex workers’ coping with various traumas, through connecting with similar others can be translated into social capital.
Coping with painful experiences	Scott et al., (2017) Husemann & Eckhardt (2019)	Learning to adapt to adversity can provide confidence in other adverse events. Sharing painful narratives with others may lead to status enhancements.
Coping with terminal illnesses	Pavia & Mason (2004)	Learning to survive one round of chemo may provide confidence that you can do it again if needed.

It would be fruitful to reinvestigate how coping capital may be converted into other forms of capital. For example, coping with ‘ridicule’ to become a member of an adolescent group (Wooten, 2006) can be seen as developing coping capital that allows the person to socialize and enter into the group and therein learn about the negative consequences of nonconformity. Questions of concern in such inquiries include: In what ways can facing ridicule lead to a resource with benefits? Is it ‘capital’ (in the sense of Bourdieu’s capital)? Is the process of dealing with ridicule (i.e., acquiring coping capital) purposeful or unintentional? If facing ridicule does generate coping capital, what other forms of capital does it translate into? Another context of interest is that of immigrants’ coping with acculturation stress. Dealing with acculturation stress by learning to fit in can help immigrants from a developmental perspective, such as in improving their social behaviors (Schönpflug, 1997). Similar to the possible research inquiry and reinquiry questions posed above in the context of ridicule confronting, it might be interesting to examine whether and how coping with acculturation stress can be regarded as a form of capital, and if so, what other forms of capital it translates into. Both of these examples—coping with ridicule and acculturation stress—can also be regarded as ways of building social capital in Bourdieu’s framework.

Another potential traumatic phenomenon arises with the use of dating apps. The use of these apps is not without risks. Risks might include security and privacy issues connected to app configuration, psychological consequences from problematic app use, antisocial conduct in applications, and unhealthy and obsessive body image worries (Castro & Barrada, 2020). In this regard, research on dating apps shows how learning from past (or others’) mistakes leads to coping better with current and future dating app experiences. Wong et al., (2020), for instance, used the knowledge of past safety measures employed by dating app users to cope with personal safety problems, privacy issues, and sexual health when using dating apps. The knowledge gathered about prior coping strategies aided in their ‘needs assessment.’ Wong et al.’s (2020) study designed an intervention for safer dating app usage using this resource and using novel techniques such as crowdsourcing and peer-led initiatives. The benefits reaped in this case were on a macro-level. Even at the micro-level of an individual’s experiences, coping strategies used in dating app use can provide benefits. Sobieraj & Humphreys (2021), for example, show that women employ coping strategies such as swiping together to mock males. This not only fosters “gender solidarity” but also allows a woman player to demonstrate to her friends that she is skilled at playing dating games, boosting her self-esteem (Sobieraj & Humphreys, 2021, p. 13). Quite possibly, this may lead to symbolic and social capital.

Apart from reexamining the generation of coping capital and its potential transformation into other forms of capital among various consumer groups, such as those coping with illiteracy, homelessness, stigma, painful experiences, and terminal illnesses, as illustrated in Table 2, it may be worthwhile to investigate the emergence of distinct types of (dis)similar capitals within consumer groups like those mentioned above. Tapp & Warren (2010) suggest, “[t]he need for people to compete and compare by deploying capital in fields is not [just] socially attractive but a major, even dominant theme of some lives” (p. 219). Therefore, another possible focus for further research is the fields or settings (e.g., social, cultural, or political) in which coping capital is deployed to gain better social positioning in the respective field. However, in all these examples, empirical reinvestigations are needed to first demonstrate the existence of coping capital and then to determine if and how it translates into other forms of capital.

## Theoretical questions for future research

The previous section considers various consumer coping phenomena. At the same time, there are some important theoretical research questions in need of future study. We identify five of them here.

1. Can these coping skills be taught without having to learn “the hard way”? One example is training at the second author’s university to reduce future susceptibility to cyber-threats such as phishing, tele-scams, and other online security threats. After a mandatory training program and successfully passing quizzes, a false phishing attempt is made some time in the two weeks after passing the online training, not only to see whether the employee is able to apply their book learning, but more importantly to see whether they fall victim to the phishing attempt. In either case it is hoped that the coping accrued in responding will sensitize and inoculate them against real phishing attempts. Previous phishing and security warning experiences have been shown in empirical studies to improve information security awareness, resulting in a considerable increase in coping efficacy (Jaeger & Eckhardt, 2021). New research avenues related to coping with cybersecurity could emerge based on our concept of coping capital as the (un)intentional resource accumulation oftentimes existing in an embodied form as dispositions.
2. A second theoretical issue involves the extent to which coping skills learned in one domain can be transferred to different situations. Notably, psychological coping resources play an essential role as determinants of athletic performance in athletic settings where athletes encounter a variety of new physical and psychological obstacles (Christensen & Smith, 2018). Can athletic coping capital learned to avoid getting “psyched out” in a competition be applied to job searches in which job applicants are in a room while waiting to be interviewed for a job opening? This theoretical possibility also relates to Bourdieu’s discussion of transferability as a central characteristic of capital, which we discuss in our literature review section. 
3. Consumers employ various coping techniques to alleviate post-purchase regret, according to current research which has examined their post-purchase experiences and regret, as well as impulsive buying (Cornish, 2020). Also worthy of investigation is the issue of whether consumers learn to reign in their impulsive desires if they have had prior impulsive purchase regrets. Is this embodied in the sense that it manifests as long-term mental dispositions to restrain or avoid impulsive purchases (Bourdieu, 1986)? While this avenue of developing coping capital is broadly recommended, there is a competing theory that suggests that it is easier to develop these skills in coping (i.e., coping capital) by occasionally “letting of the steam” of patience through small indulgences like chocolate while out practicing thrifty frugality on behalf of the family (Miller, 1998). The two theories might be pitted against one another in a retrospective or longitudinal study. The size of the indulgence and the size of prior regret may be relevant as well.
4. Is it possible that those with obesity (Puhl & King, 2013), severe facial disfigurements (Henley, 2021), and other handicaps and who have suffered bullying and unwarranted inferences that they also must have mental impairments, have better coping skills to deal with setbacks in other areas of adult life (e.g., denied loan requests, journal rejections and negative reviews, failure in job hunts)? This assumes that they found positive coping skills to deal with such injustices without giving up. Such investigations are expected to contribute to the discourses on growth and empowerment (e.g., Henry, 2004; Gutiérrez, 1994).
5. Within a culture and social class backgrounds, is there more resilience and persistence in the face of difficulties from those who have experienced more difficult circumstances (e.g., who report more bullying as a child, having had more family changes, more medical difficulties, or less success in sports or grades); is there evidence of stronger coping capital as an adult? Exploring the presence of positive coping abilities, as in this example, may have implications from a developmental perspective over a person’s entire lifespan (Burrow & Hill, 2020).

These examples show how we might better understand the mechanisms of coping capital through examining the vertical transfer (reencountering the same potential source of trauma) as well as lateral transfer (to different challenging circumstances) of coping capital. It may be that the benefits of coping capital work differently in the two types of situations. Vertical transfer may involve learned behavior patterns while horizontal (lateral) transfer may involve learned internal responses such as patience and confidence. In both cases as further work is conducted our understanding of coping capital will increase.

## A word of caution

Although we propose that certain potential phenomena should be examined or reexamined in considering coping capital and present some theoretical pathways in the preceding sections, we have qualms about advocating the initiation of an empirical project using our own or any other a priori theory. This disclaimer is intended so that we are not constrained by rigid standards and guidelines to adopt or adapt any a priori theory that can restrict the use of our imagination. Starting with a singular priori theory tends to shape data collection and it becomes an attempt to apply an existing framework in a way that blinds the researcher to other possible interpretations of the data. It shapes the questions asked and the things observed while blinding us to other possibilities. As Belk & Sobh (2019) demonstrate in examining Wilk’s theory of binging as one alternative to an emergent masking and bluffing theory, ultimately the proof is in the eating of the pudding. Alternative theoretical lenses are pitted against the emergent theory in accounting for the data. Alternatively, if the research had begun with a singular theoretical framework, the data to assess these other possibilities might well not have been collected at all. That said, rejecting a priori theory-aided research does not at all preclude pitting prior theories against existing theories in interpreting findings.

The above point is illustrated in Belk & Sobh (2019). They begin by looking for surprises, anomalies, and puzzles in the data. In the illustration in that paper, the authors show how a dozen prior theories might help understand the puzzle in Newell’s (2012) book. They are pitted against Newell’s bluffing theory that he derived with abductive reasoning. Belk & Sobh (2019) also offer criteria for making such comparisons and recognize that an existing theory might well come out best. It is just that starting with an a priori theory for a study affects the data gathered, the analysis employed, and the range of alternatives considered if any. Like Daniel Simon’s “monkey business illusion,” when you are looking for one thing, you may well fail to spot “the gorilla in the room.” Thus, we affirm that avoiding theory-aided research in pursuit of original theory is not an impediment to using existing theory. In fact, it provides a more rigorous means of assessing multiple possible existing theories. Those using a grounded theory/abductive reasoning approach should be well-read in various potentially applicable theories in various related fields. They could well end up employing one or more existing theories (including our own findings) in their interpretation of the data; but not before attempting an open approach capable of generating original theory. For a counterpoint arguing in favor of theory-aided research, see Dolbec et al.(2021). There is also a forthcoming *Marketing Theory* “Conversation” between these three authors and Belk.

## Conclusions

Returning to the research questions stated in the introduction, we have shown that coping with adversity can be conceived of as a resource, which we call coping capital. Coping capital is relational, accumulative, and translatory similar to Bourdieu’s notion of cultural capital. The application of Bourdieu’s idea of capital to extend our understanding of the coping phenomena is a significant strength of this study. And we believe that one of the most significant outcomes to come from this research would be the evidence about the existence of coping capital and how it reshapes into various other types of capital in diverse settings.
